# Effect of bedding dip angle and dynamic load on spatial variation of microscopic failure stress of shale

**DOI:** 10.1038/s41598-023-30519-w

**Published:** 2023-03-08

**Authors:** Miaomiao Wang, Xiaozhou Shao, Xiaohua Pan

**Affiliations:** 1 School of Highway, Chang’an University, Xi’an, 710064 China; 2 College of Energy Resources, Chengdu University of Technology, Chengdu, 610059 China; 3 PetroChina Changqing Oilfield Company, Xi’an, 710018 China; 4 School of Earth Science and Engineering, Nanjing University, Nanjing, 210023 China

**Keywords:** Solid Earth sciences, Energy science and technology, Engineering

## Abstract

Physico-mechanical properties of shale are important parameters in evaluating the stability of potential wellbore and the design of hydraulic fracturing, which are primarily affected by their non-uniform spatial distribution of the microscopic physical–mechanical properties at particle scale. A series of constant strain rate experiments and stress-cycling experiments on shale specimens with different bedding dip angles were conducted to have a comprehensive understanding of the effect of the non-uniform distribution of microscopic failure stress on macroscopic physico-mechanical properties. According to the experimental results and Weibull distribution, we find that bedding dip angle and the dynamic load applying type affect the spatial distributions of microscopic failure stress. The values of crack damage stress (*σ*_cd_),* σ*_cd_/*σ*_ucs_ (peak stress), *ε*_cd_ (strain at crack damage stress), Poissons' ratio (*ν*), elastic strain energy (*U*_e_) and dissipated energy (*U*_irr_) of the specimens with more uniform distribution of microscopic failure stress are overall higher, while *ε*_ucs_ (peak strain)/*ε*_cd_ and elastic modulus (*E*) are lower. The dynamic load enables the spatial distributions of microscopic failure stress trend to be more homogeneous prior to the final failure with the increment of *σ*_cd_/*σ*_ucs_, *ν*, *U*_e_ and *U*_irr_ and the decrement of *E*.

## Introduction

Shale is one of the various rock types encountered when exploring and drilling for petroleum and shale gas as well as during civil engineering project construction process^1–6^. Its macroscopic physico-mechanical properties are important parameters in evaluating the stability of potential wellbore and the design of hydraulic fracturing, etc. The effects of microstructure on macroscopic physico-mechanical properties of shale have been one of the major research topics in recent years ^5,7–15^. These studies indicate that the heterogeneity of the microstructure is an intrinsic composite characteristic of the shale, which is made up of various mineralogical components, particle-sizes, porosities, cracks, etc. The composite heterogeneous microstructure induces non-uniform distribution of microscopic physico-mechanical properties at particle scale, significantly affecting rock progressive failure process and macroscopic physico-mechanical properties^16–26^. Therefore, it is important to investigate the relationship between the microscopic and macroscopic physico-mechanical properties of shales. However, due to the characteristics of the random distribution, the microscopic physico-mechanical properties are difficult to be quantified by analytical methods or measured by experiments as other rock and rock-like materials^27^.

**Figure 1:**
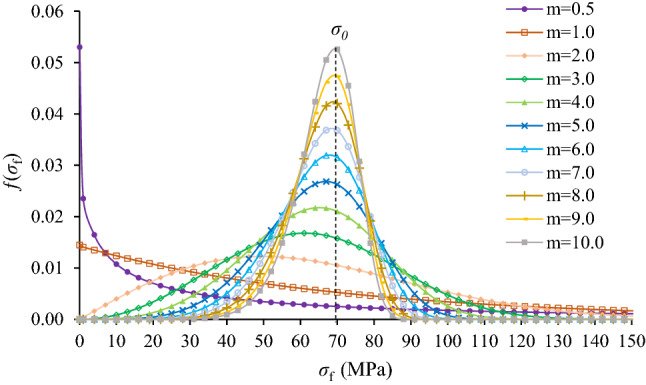
Weibull probability distribution of failure strength for different homogeneity index *m.*

Weibull distribution has been used widely to model the statistical distribution of the microscopic physical–mechanical properties of rock or rock-like materials induced by the multiscale material heterogeneities^28–32^. The microscopic physical–mechanical properties, such as element failure stress (hereinafter referred to as failure stress), *σ*_f_, are assumed to be randomly distributed throughout the specimen elements following a Weibull probability distribution function:

1 $$f\left({\sigma }_{f}\right)=\frac{m}{{\sigma }_{0}}{\left(\frac{{\sigma }_{f}}{{\sigma }_{0}}\right)}^{m-1}{e}^{-{\left(\frac{{\sigma }_{f}}{{\sigma }_{0}}\right)}^{m}}$$

where *σ*_0_ is the mean failure strength of the elements of the specimen, *m* is the homogeneity index of the material. For given *σ*_0_, an infinitely high *m* value corresponds to a uniform distribution of failure strength, whereas a broad distribution of failure strength is associated with a relatively low *m* value^33,34^. A case of Weibull strength distribution for different homogeneity index *m* is shown in Fig. 1. It indicates that the heterogeneity of failure stress can be characterized by the Weibull distribution with a reasonable homogeneity index *m*.

In previous numerical modelling studies, Weibull distribution with various *m* values has been used as a promising statistic model to characterize the spatial variation of the microscopic physico-mechanical properties of rock and rock-like materials^35–39^. One of the challenges of using Weibull distribution is to obtain the *m* value practically. In these studies, the *m* values were normally obtained through the Linear Least Squares (LLS) technique, which involves the ranking of *σ*_f_ of the elements of the modelled specimen in ascending order^40^. In order to assess the spatial variation of the microscopic physical–mechanical properties of rock specimen in laboratory scale, Pan et al.^41^ proposed an experimental method for obtaining the *m* value via macroscopic physico-mechanical parameters.

What’s more, dynamic cyclic loading is an important loading type for shales near the wellbore surface and hydraulic fractured surface which are under conditions of uniaxial pressure or low confining pressure^42^. The objective of this paper is to investigate the effect of the non-uniform distribution of microscopic failure stress on macroscopic physico-mechanical properties of shales subjected to uniaxial dynamic pressures based on Weibull distribution.

## Methodology

**Figure 2:**
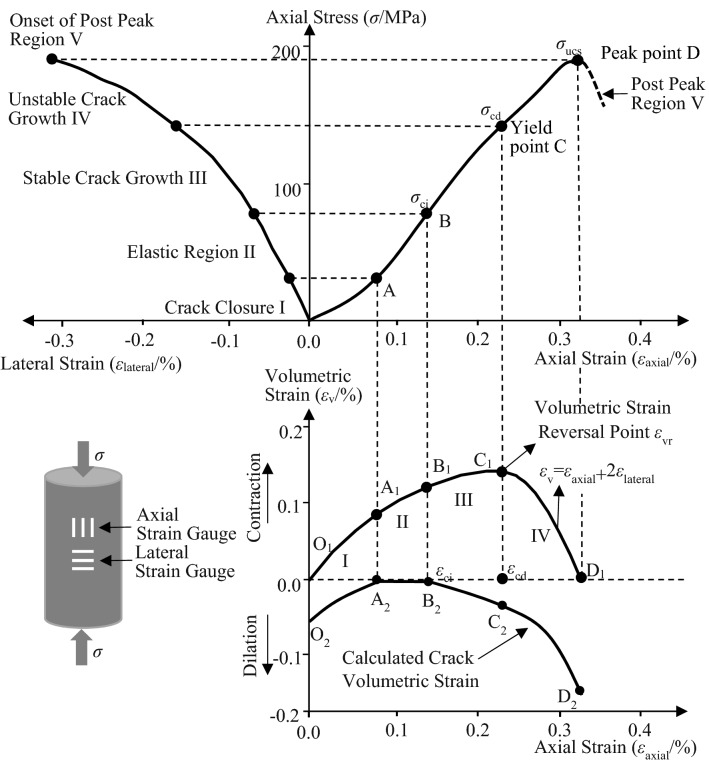
Stress–strain diagram showing the steps of crack development and relationship with volumetric strain-axial strain curve (modified after Martin and Chandler^43^).

Obtaining the value of *m* practically is a critical issue to characterize the heterogeneous distribution of *σ*_f_ of shale specimens using Weibull distribution. Pan et al.^41^ constructed a quantitative relationship between *m* value and strain ratio (*ε*_ucs_/*ε*_cd_) of specimen subjected to uniaxial compression loading. As shown in Fig. 2, peak strain *ε*_ucs_ is the strain at peak stress *σ*_ucs_ (point D), crack damage strain *ε*_cd_ is the strain at crack damage stress *σ*_cd_ (point C). The value of* m* can be determined after the *ε*_ucs_/*ε*_cd_ has been estimated through experimental results. It is well known that *ε*_ucs_ can be directly determined from the stress–strain curve while it is difficult for *ε*_cd_. Therefore, volumetric strain reversal point, *ε*_vr_, (point C_1_ in volumetric strain-axial strain curve as shown in Fig. 2) is used to estimate the *σ*_cd_. The quantitative relationship between *m* and *ε*_ucs_/*ε*_cd_ is presented as follows.

### Relationship between the homogeneity index m and strain ratio ε_ucs_/ε_cd_

For a cylindrical core specimen of rock and rock-like material in laboratory scale, the *σ*_f_ of each element was suggested to obey a Weibull distribution to represent the inherent heterogeneity of failure strength distribution. When the specimen was subjected to uniaxial compression load, a quantitative relationship between *m* (*m* ≥ 1) and *ε*_ucs_/*ε*_cd_ was constructed based on two-dimensional renormalization group theory by Pan et al.^41^ as follows.

2 $$\frac{{\varepsilon }_{\mathrm{ucs}}}{{\varepsilon }_{\mathrm{cd}}}={\left[-\frac{1}{m\mathit{ln}\left(1-{A}_{*}+{B}_{*}^{\frac{1}{{C}_{*}^{m}-{D}_{*}}}\right)}\right]}^\frac{1}{m}$$

where A_*_ = 1.004079, B_*_ = 0.768013, C_*_ = 1.603054, and D_*_ = 1.131608.

However, the relationship was not established as 0.5 ≤ *m* < 1. Based on Eq. 8 presented in Pan et al.^41^, A_*_, B_*_, C_*_ and D_*_ for 0.5 ≤ *m* < 1, is extracted by the trust region regression method^44^, which are 1.036838, 0.219326, 3.210927 and 0, respectively.

The analysis based on the results indicates that *ε*_ucs_/*ε*_cd_ gradually decreases as *m* increases from 0.5 to 30. Thus, the *m* value can be obtained once the *ε*_ucs_/*ε*_cd_ is determined.

### Determination of the crack damage strain ε_cd_

Volumetric strain (*ε*_v_) is a pervasive volumetric property of the rock and rock-like material^45^. For a cylindrical specimen subjected to axial loading and under small strain, the volumetric strain is given by Martin and Chandler^43^:

3 $${\varepsilon }_{\mathrm{v}}={\varepsilon }_{\mathrm{axial}}+2{\varepsilon }_{\mathrm{lateral}}$$

An example of relationship between axial, lateral strain versus axial stress curve and volumetric strain versus axial strain curve in uniaxial compression is given in Fig. 2. The axial strain level where the volumetric strain reversal occurs at point C_1_ (volumetric strain reversal point *ε*_vr_) marks the beginning of Region IV and represents the onset of unstable crack growth at point C^43,46^. The volumetric strain reversal occurs at point C_1_ can be directly obtained in the curve. Therefore, the *ε*_cd_ can be estimated using the same strain level of *ε*_vr_.

## Experiments

In this study, constant strain rate experiments and stress-cycling experiments were both performed in a RTR-2000 triple-axis dynamic testing system. It was manufactured by GCTS Company, USA, for physico-mechanical properties testing of rocks with a maximum axial load of 2000 KN and a maximum confining pressure of 140 MPa. Constant strain rate experiments were carried out to estimate the* σ*_ci_ (crack initiation stress as shown in Fig. 2 at point B),* σ*_cd_ and *σ*_ucs_ of shale specimens with the same bedding dip angle which would be tested under dynamic loading afterwards. Stress-cycling experiments were conducted to investigate the effect of dynamic loading on heterogeneous distribution of *σ*_f_ and physico-mechanical properties.

### Specimen preparation

**Figure 3:**
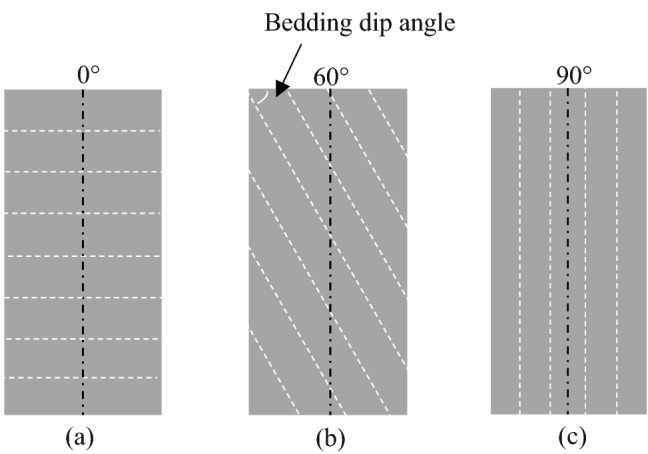
Plots of shale specimens cored from a shale block with different bedding dip angles (*θ*): (**a**) 0°, (**b**) 60° and (**c**) 90°.

Shale specimens with three types of bedding dip angles were cored through a shale block with different drilling angles. The block collected from Lower Silurian Longmaxi Formation of Sichuan basin in Chongqing, China, was composed of quartz, feldspar, pyrite, clay, calcite, mica, and a connected low porosity and low permeability. As shown in Fig. 3, three types of bedding dip angles (*θ* = 0°, 60° and 90°) made with specimen end surfaces were conducted. The specimens of each type of bedding dip angle were prepared in quintuplicates, which had been standardized by International Society for Rock Mechanics standards^47^. Each specimen had a 50 mm in diameter and a length to diameter ratio of 2, and the end surfaces were flat with an accuracy of 0.02 mm. The mean acoustical properties for three types of shale specimens with different bedding dip angles were presented in Table 1. It indicates that the difference of micro-structures among the specimens were dominated by the bedding dip angles.

**Table 1:** Mean acoustical properties for shale specimens with three types of bedding dip angles.

Bedding dip angle *θ* (°)	0	60	90
*P*-wave velocity (m/s)	4091	4362	4563

### Testing procedure

**Figure 4:**
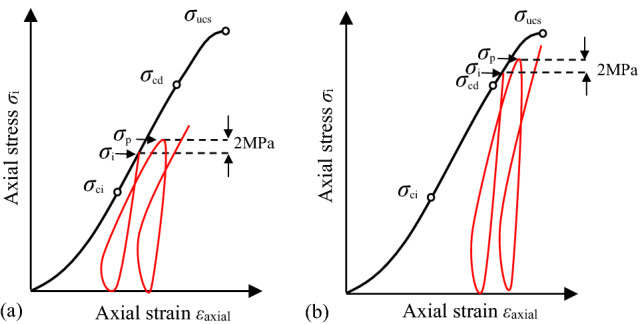
Plots of two types of dynamic cyclic loads used in experiments. Loading–unloading procedures start after an initial stress *σ*_i_ has been applied to the value (**a**) larger than *σ*_ci_ and smaller than *σ*_cd_, and (**b**) larger than *σ*_cd_ and smaller than *σ*_ucs_. The subsequent peak stress (*σ*_p_) per cycle had a 2 MPa increment until final failure occurred.

The quintuplicate specimens for each type of bedding dip angle were separated into five groups named A, B, C, D and E, respectively. Specimens of group A, B and C were applied at a constant strain rate of 6.0 × 10^–4^/s. The *σ*_ucs_ was directly obtained from the stress–strain curve, the *σ*_cd_ was estimated using the method presented in Sect. "Determination of the crack damage strain ε_cd_" corresponding to *ε*_vr_, and the *σ*_ci_ can be identified from the point where the horizontal section of calculated crack volumetric strain-axial curve ends (point B_2_ in Fig. 2)^43^. Specimens of group D and E were applied with two types of dynamic cyclic load, respectively, which have different stress value of the first-time unloading as shown in Fig. 4. For specimens of group D, the first-time unloading was carried out until the axial stress (*σ*_i_) exceeded the *σ*_ci_ but lower than the *σ*_cd_ as shown in Fig. 4a. The determination procedure of *σ*_i_ of the specimen of group D with the 0° bedding dip angle was presented as an example. Firstly, the mean values of *σ*_ci_,* σ*_cd_ and *σ*_ucs_ of specimens of groups A, B and C (*θ* = 0°) were 25.93, 59.93 and 98.31 MPa (see Table 2), respectively. Therefore, 35.00 MPa (larger than *σ*_ci_ = 25.93 MPa and smaller than *σ*_cd_ = 59.93 MPa) with *σ*_i_ /*σ*_ucs_ = 0.36 (larger than *σ*_ci_ /*σ*_ucs_ = 0.26 and smaller than *σ*_cd_ /*σ*_ucs_ = 0.61) was determined as the *σ*_i_ of the first-time unloading for specimen of group D (*θ* = 0°). However, the *σ*_ucs_ (128.16 MPa) of specimen of group D (*θ* = 0°) was much higher than the mean value of that of specimens of groups A, B and C (*θ* = 0°) as affected by the dynamic cyclic loads. As a result, the values of estimated *σ*_i_ (35.00 MPa) and *σ*_i_/*σ*_ucs_ (0.27) were much lower than the *σ*_ci_ (48.70 MPa) and *σ*_ci_/*σ*_ucs_ (0.38) of specimen of group D (*θ* = 0°). Secondly, a new test with a new specimen of group D_2_ (*θ* = 0°) was thus carried out with higher *σ*_i_ (75.00 MPa). Following the same procedure, the reasonable values of *σ*_i_ of specimens of group D for *θ* = 60° and *θ* = 90° were determined. For specimens of group E, the axial stress *σ*_i_ of the first-time unloading was between the *σ*_cd_ and *σ*_ucs_ as shown in Fig. 4b. The values of *σ*_i_ were estimated by the same procedure as those used in specimens of group D. Subsequently, peak stress (*σ*_p_) of each cycle of the two types of dynamic cyclic loads both had a 2 MPa increment until final failure occurred.

**Table 2:** Experimental results of shale specimens of groups A, B, C that were obtained from the constant strain rate experiments and corresponding results of shale specimens of groups D and E.

Bedding dip angle	Specimen No	*σ*_ucs_ (MPa)	*σ*_ci_ (MPa)	*σ*_cd_ (MPa)	*σ*_ci_/*σ*_ucs_	*σ*_cd_/*σ*_ucs_	*σ*_i_ (MPa)	*σ*_i_/*σ*_ucs_
*θ* = 0°	Group A	104.65	26.92	62.37	0.26	0.60	–	–
	Group B	92.11	20.43	47.74	0.22	0.52	–	–
	Group C	98.16	30.43	69.69	0.31	0.71	–	–
	Group D Group D_2_	128.16 159.37	48.70 50.37	89.71 96.92	0.38 0.32	0.70 0.61	35.00 75.00	0.27 0.47
	Group E	96.97	34.94	80.77	0.36	0.83	81.00	0.84
*θ* = 60°	Group A	57.98	18.07	34.98	0.31	0.60	–	–
	Group B	53.00	15.70	28.04	0.30	0.53	–	–
	Group C	57.92	16.82	44.45	0.29	0.77	–	–
	Group D	49.51	17.75	44.52	0.36	0.90	32.00	0.65
	Group E	71.98	21.77	46.29	0.30	0.64	60.00	0.83
*θ* = 90°	Group A	81.79	24.76	63.29	0.30	0.77	–	–
	Group B	80.82	24.36	46.58	0.30	0.58	–	–
	Group C	72.81	6.89	39.71	0.09	0.55	–	–
	Group D	133.96	39.19	83.91	0.29	0.63	40.00	0.30
	Group E	87.71	34.64	43.07	0.39	0.49	70.00	0.80

## Results

### Mechanical properties of shale specimens of groups A, B and C

**Figure 5:**
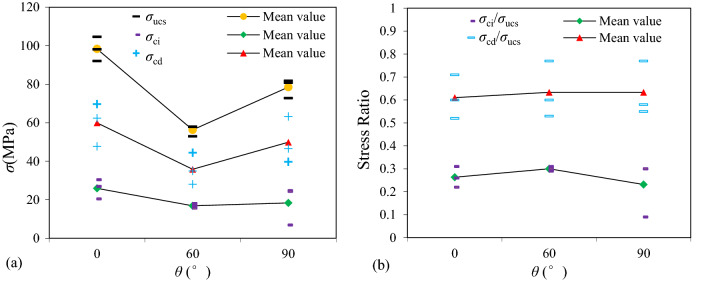
Plots of mean values of (**a**) *σ*_ucs_, *σ*_ci_, *σ*_cd_ and (**b**) *σ*_ci_/*σ*_ucs_, *σ*_cd_/*σ*_ucs_ of shale specimens of groups A, B and C for each bedding dip angle.

Table 2 first presents the experimental results of the shale specimens of groups A, B and C with different bedding dip angles (*θ* = 0°, *θ* = 60° and *θ* = 90°) obtained from the constant strain rate experiments, i.e., *σ*_ucs_, *σ*_ci_, *σ*_cd_, *σ*_ci_/*σ*_ucs_, *σ*_cd_/*σ*_ucs_, which were used to estimate the *σ*_i_ of specimens of group D and E. Figure 5 shows the mean values of *σ*_ucs_, *σ*_ci_, *σ*_cd_, *σ*_ci_/*σ*_ucs_ and *σ*_cd_/*σ*_ucs_ of shale specimens of groups A, B and C for each dip angle. Figure 5a indicates that the mean values of *σ*_ci_, *σ*_cd_, *σ*_ucs_ of shale specimens for *θ* = 90° were larger than that for *θ* = 60° but smaller than the *θ* = 0°, i.e., the mean values of *σ*_ucs_ for *θ* = 90°, *θ* = 60° and *θ* = 0° were 78.47, 56.30, 98.31 MPa, respectively. Figure 5b indicates that the mean values of* σ*_cd_/*σ*_ucs_ did not increase significantly as bedding dip angle increased which were 061, 0.63 and 0.63, while the mean values of *σ*_ci_/*σ*_ucs_ increased slightly as bedding dip angles increased from 0° (0.26) to 60° (0.30) and decreased as bedding dip angles increased to 90° (0.23).

### Variation of physico-mechanical properties of shale specimens of groups D and E

**Figure 6:**
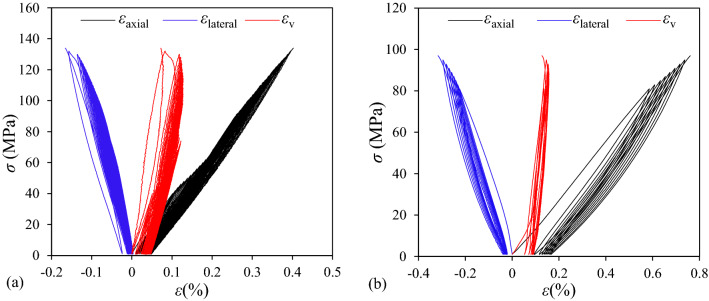
The stress–strain curves of specimens of (**a**) *θ* = 90° in group D and (**b**) *θ* = 0° in group E.

The typical stress–strain curves of specimens of *θ* = 90° in group D and *θ* = 0° in group E were shown in Fig. 6. Except the estimated *σ*_i_ and *σ*_i_/*σ*_ucs_, *σ*_ucs_, *σ*_ci_, *σ*_cd_, *σ*_ci_/*σ*_ucs_ and *σ*_cd_/*σ*_ucs_ were also presented in Table 2. *σ*_ci_ of group D was determined from the first cycle loading and *σ*_cd_ from the first cycle loading that the *σ*_p_ exceeded the crack damage stress using the method presented in Sect. "Determination of the crack damage strain ε_cd_", while the *σ*_ci_ and *σ*_cd_ of group E was determined from the first cycle loading as the initial loading was larger than the crack damage stress. *σ*_ucs_, *σ*_ci_, *σ*_cd_ of specimens of groups D and E with *θ* = 0° were both larger than that of *θ* = 90°, and *θ* = 60° were the smallest, i.e., *σ*_ucs_ of groups D for *θ* = 0°,* θ* = 90° and *θ* = 60° were 159.37, 133.96 and 49.51 MPa, respectively, which were in descending order. For specimens of dip angles of *θ* = 0° and *θ* = 90°, *σ*_ucs_, *σ*_ci_, *σ*_cd_ of of group D were larger than that of group E, while for specimens of dip angles of *θ* = 60°,* σ*_ucs_, *σ*_ci_, *σ*_cd_ of of group D were smaller than that of group E. For example, *σ*_ucs_ of groups D and E of *θ* = 0°were 159.37and 96.97 MPa, for *θ* = 90° were 133.96 and 87.71 MPa, while for *θ* = 60° were 49.51 and 71.98 MPa.

**Figure 7:**
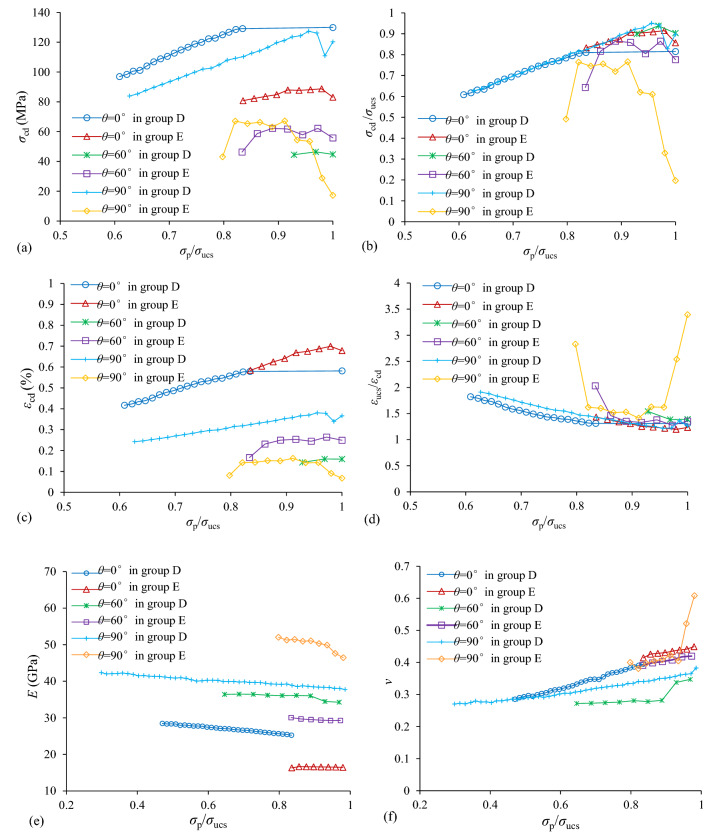
Evolution of (**a**) *σ*_cd_, (**b**) *σ*_cd_/*σ*_ucs_, (**c**) *ε*_cd_, (**d**) *ε*_ucs_/*ε*_cd_, (**e**) *E* and (**f**) *ν* for different shale specimens as *σ*_p_/*σ*_ucs_ in ascending order.

Figure 7 presented the experimental results of each cycle loading of shale specimens of groups D and E with different bedding dip angles, including *σ*_cd_, *σ*_cd_/*σ*_ucs_,* ε*_cd_,* ε*_ucs_/*ε*_cd_, elastic modulus (*E*), Poissons’ ratio (*ν*). Figure 7a and c indicate that the *σ*_cd_ and *ε*_cd_ of all specimens of group D with various dip angles increased as *σ*_p_/*σ*_ucs_ increased before final failure as well as descending order phenomenon was observed at final failure. What’s more, for the same loading type, the values of *σ*_cd_ and *ε*_cd_ of specimens of group D with *θ* = 0° were the largest while the *θ* = 60° were the smallest. For specimens of group E, the values of *σ*_cd_ and *ε*_cd_ of *θ* = 0° were larger than those of *θ* = 60° and *θ* = 90°. Figure 7b and d indicate that the* σ*_cd_/*σ*_ucs_ and* ε*_ucs_/*ε*_cd_ of all specimens of group D with various dip angles were almost the same, which were in ascending and descending orders as *σ*_p_/*σ*_ucs_ increased, respectively. For specimens of group E, Fig. 7b shows that the values of *σ*_cd_/*σ*_ucs_ of various bedding dip angles decreased significantly at final failure especially for* θ* = 90°, and the values of *θ* = 0° were largest as well as *θ* = 90° were smallest. For the *ε*_ucs_/*ε*_cd_ of specimens of group E, values of *θ* = 90° decreased at initial loading cycles and increased significantly at final failure, which were the largest and that of *θ* = 0° were the smallest.

The *E* and *ν* of each cycle loading were obtained here by Eqs. (4) and (5), respectively.

4 $$E=\frac{{\sigma }_{\mathrm{p}}}{{\varepsilon }_{\mathrm{axial}}-{\varepsilon }_{\mathrm{axial}}^{\mathrm{irr}}}$$

5 $$\nu =-\frac{{\varepsilon }_{\mathrm{lateral}}-{\varepsilon }_{\mathrm{lateral}}^{\mathrm{irr}}}{{\upvarepsilon }_{\mathrm{axial}}-{\varepsilon }_{\mathrm{axial}}^{\mathrm{irr}}}$$

where, ${\varepsilon }_{\mathrm{axial}}^{\mathrm{irr}}$ and *ε*_axial_ was the irrversible and total axial strain at the peak stress *σ*_p_ in each loading cycle respectively; ${\upvarepsilon }_{lateral}^{irr}$ and *ε*_lateral_ was the irrversible and total lateral strain at the peak stress *σ*_p_ respectively. These definiation correlated with the elatic rebound during unloading stage were more appropriate to the measurements of the elastic parameters for cyclic loading tests^48^. The evolutions of *E* and *ν* of each cycle loading for specimens in group D and E as a function of the normalized stress level (*σ*_p_/*σ*_ucs_) in each loading cycle were presented in Fig. 7e and f. Figure 7e indicates that the *E* for all specimens decreased as dynamic load increased until final failure. For the same loading type, the values of *E* of specimens with *θ* = 0° were the smallest while those with *θ* = 90° were the largest. Figure 7f shows that the *ν* were in ascending order as the dynamic load increased. For the loading type of group D, the values of *ν* of specimens with *θ* = 90° were larger than those of *θ* = 60° and smaller than those of *θ* = 0°. For the loading type of group E, the values of *ν* of specimens among *θ* = 0°, *θ* = 60° and *θ* = 90° were almost the same except the values of *ν* of specimens with *θ* = 90° increased significantly at last two loading cycles.

**Figure 8:**
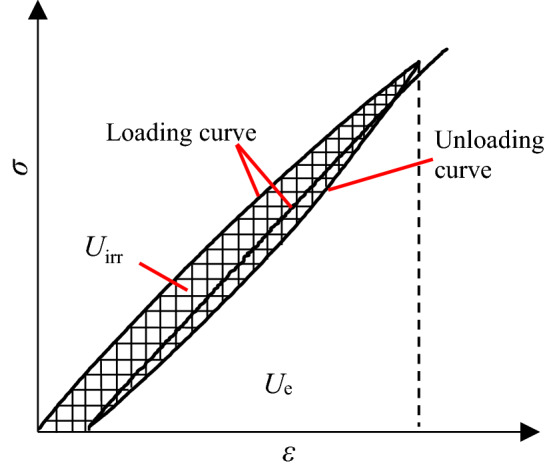
Relationship between elastic strain energy (*U*_e_) and dissipated energy (*U*_irr_) of rock mass unit.

**Figure 9:**
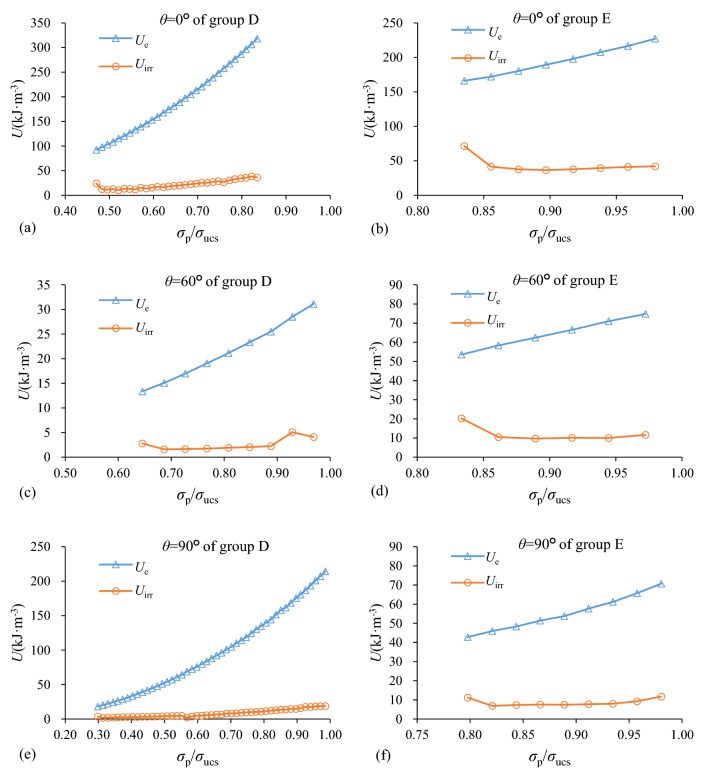
Evolution of *U*_e_ and *U*_irr_ of shale specimens of two groups with different dip angles as *σ*_p_/*σ*_ucs_ in ascending order: (**a**) *θ* = 0° of group D; (**b**) *θ* = 0° of group E; (**c**) *θ* = 60° of group D; (**d**) *θ* = 60° of group E; (**e**) *θ* = 90° of group D; (**f**) *θ* = 90° of group E.

During cycle loading process, if it assumed that there was no heat exchange, the compression energy was transferred to elastic strain energy (*U*_e_) and dissipated energy (*U*_irr_). *U*_e_ related to the unloading elastic modulus *E*, was defined as the work done by stress and strain, equalling to the area between the unloading curve and *ε-*axis as shown in Fig. 8. It was recoverable after unloading. *U*_irr_ was defined as the irreversible work for a loading and unloading cycle which was primarily induced by micro-crack initiation, propagation and coalescence. Figure 8 shows that it equals to the area of the shape confined by the loading, unloading curves and *ε-*axis in each loading cycle. Figure 9 indicates that the changes of *U*_e_ and *U*_irr_ of each cycle for all specimens in group D and E was a function of the normalized stress (*σ*_p_/*σ*_ucs_) in each loading cycle. *U*_e_ significantly increased as *σ*_p_/*σ*_ucs_ increased, while *U*_irr_ had a descending order at the first loading cycle as well as increased in following cycles. The mean *U*_e_ was larger than that of *U*_irr_.

### Variation of distribution of microscopic failure stress of shale specimens of groups D and E

**Figure 10:**
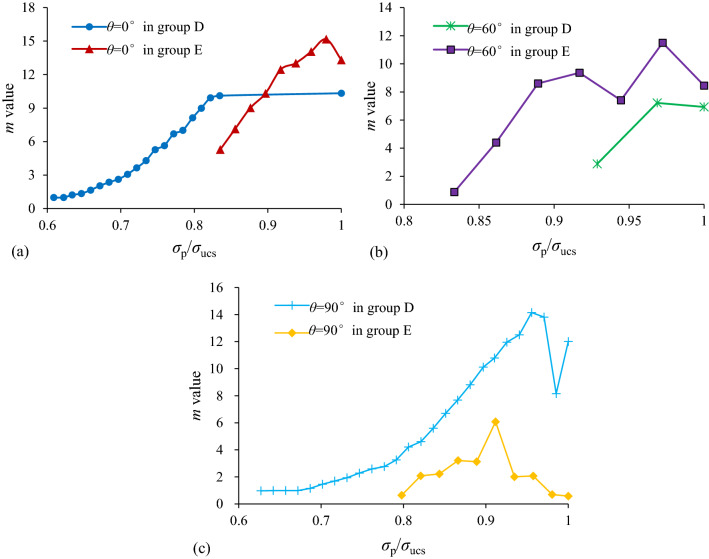
Evolutions of *m* values of shale specimens of groups D and E with different bedding dip angles as *σ*_p_/*σ*_ucs_ in ascending order: (**a**) *θ* = 0°, (**b**) *θ* = 60°, and (**c**) *θ* = 90°.

Figure 10 presents the evolutions of *m* values (determined by Eq. 2) of each loading cycle of shale specimens of groups D and E with different bedding dip angles. The *m* values of shale specimens of groups D (*θ* = 0°, *θ* = 60° and *θ* = 90°) and E (*θ* = 0° and *θ* = 60°) totally had an ascending order as *σ*_p_/*σ*_ucs_ increased as well as had a light descending order at the last one or two cycle loading before final failure. It indicates that there was a more homogeneous distribution of *σ*_f_ as *σ*_p_/*σ*_ucs_ increased. For the shale specimen of group E (*θ* = 90°), the *m* values also increased at the initial stage, but it decreased significantly in the following cycle loading.

### Final fracture morphology of specimens of groups A, B, C, D and E

**Figure 11:**
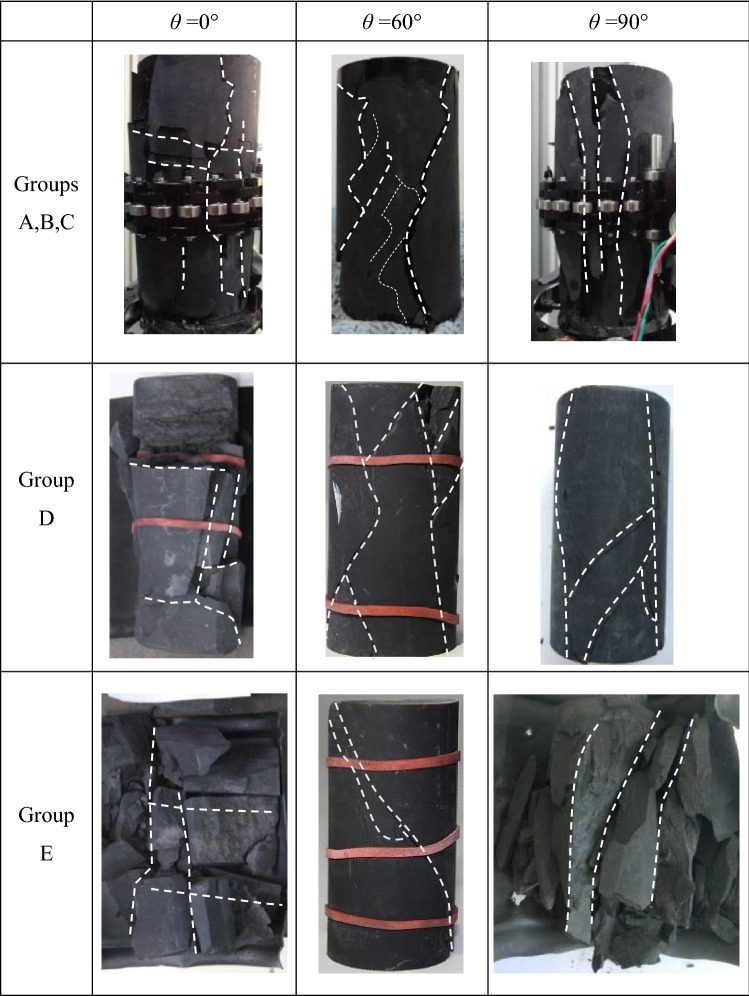
Final fracture morphology of shale specimens with different bedding dip angles during constant strain rate experiments and dynamic cyclic loading tests at different stress levels.

Figure 11 shows the typical final fracture morphology of specimens of groups A, B, C, D and E with different bedding dip angles (*θ* = 0°, *θ* = 60° and *θ* = 90°) and various loading types, which indicates that the bedding plane was the main effect affecting the final fracture morphology. For the specimens of groups A, B, C, D and E with *θ* = 0°, the fracture primarily occurred along the bedding plane in horizontal direction associated with vertical direction fracture surfaces. For the specimens of groups A, B, C, D and E with *θ* = 60° and 90°, the dip angles of the dominated fracture surface were about 60° and 90°, which were the same as that of the bedding plane.

## Discussions

### Effect of non-uniform distribution of microscopic failure stress on macroscopic physico-mechanical properties

Based on the experiment results, the effect of *m* value on physico-mechanical properties of the shale specimens subjected to dynamic load in laboratory scale were discussed.

**Figure 12:**
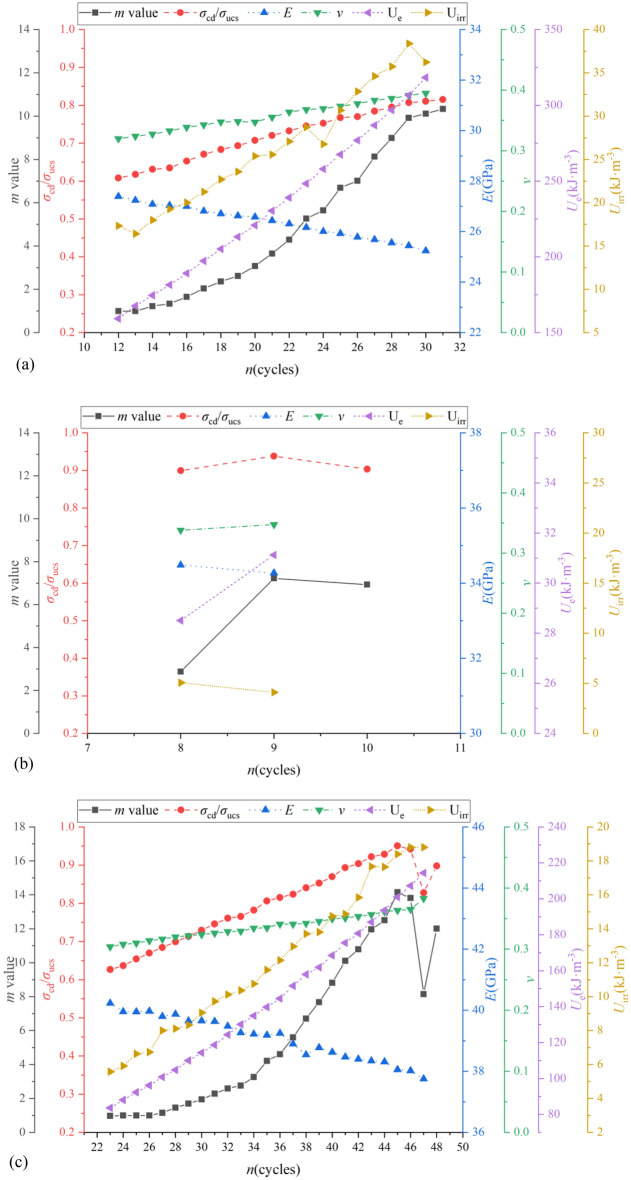
The relationship between *m* value and *σ*_cd_/*σ*_ucs_, *E*, *ν*, *U*_e_ and *U*_irr_ of shale specimens of group D with various dip angles: (**a**) *θ* = 0°; (**b**) *θ* = 60°; (**c**) *θ* = 90°.

**Figure 13:**
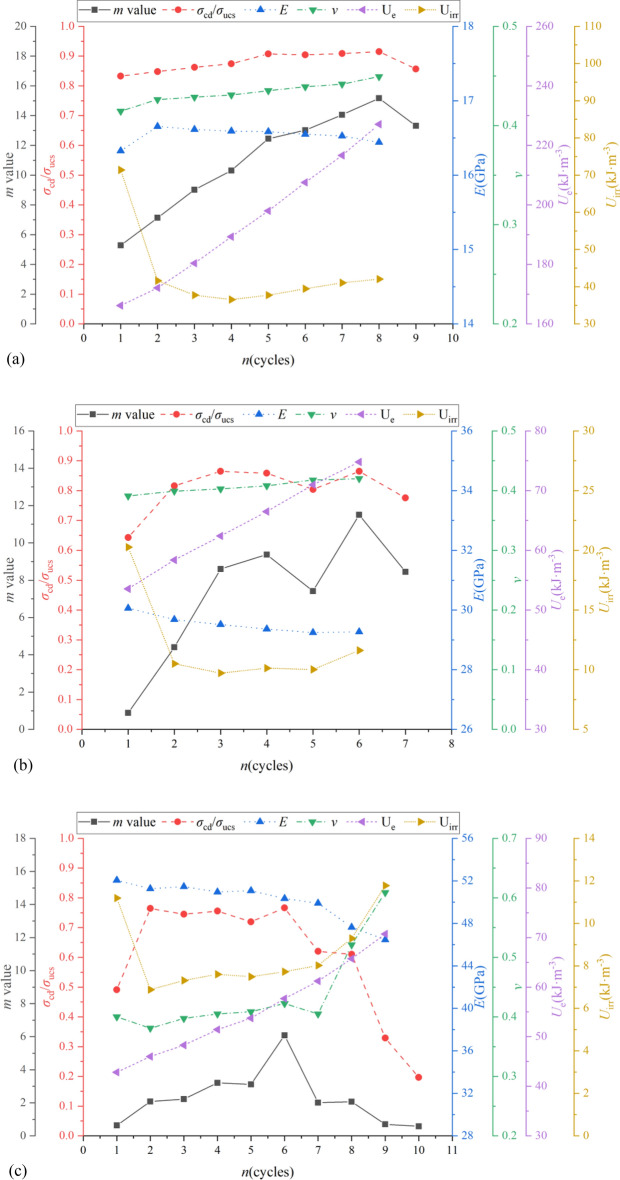
The relationship between *m* value and *σ*_cd_/*σ*_ucs_, *E*, *ν*, *U*_e_ and *U*_irr_ of shale specimens of group E with various dip angles: (**a**) *θ* = 0°; (**b**) *θ* = 60°; (**c**) *θ* = 90°.

Figures 12 and 13 shows the relationship between* m* values and *σ*_cd_/*σ*_ucs_, *E*, *ν*, *U*_e_ and *U*_irr_ of specimens of group D and E with various dip angles. The comparison results indicate that general speaking, *σ*_cd_/*σ*_ucs_, *ν*, *U*_e_ and *U*_irr_ increased as *m* values increased or there was a positive correlation between them. Conversely, *E* decreased as *m* values increased. The possible reason might be the fact that the cycles of pressure loading and unloading induce most of the low microscopic failure stresses fail first, resulting more uniform distribution of microscopic failure stresses (larger *m* values). In other words, the more times the cycles of pressure loading and unloading that were carried out, the more homogeneous the distribution of microscopic failure stresses. In can be observed from Fig. 7a that the uniform distribution of microscopic failure stresses delayed the threshold of crack damage stress (larger *σ*_cd_) during the progressive failure process with larger *σ*_cd_/*σ*_ucs_ and *ε*_cd_ (Fig. 7c). Previous study indicated more homogeneous distribution of microscopic failure stresses induced larger deformation of shale^49^. *E* (defined as the ratio of the axial stress change to axial strain produced by the stress change) thus decreased as *m* values increased. On the other hand, the homogeneous distribution of microscopic failure stresses might not only induce the large axial strain but also significantly enable great deform in the direction of the radius, resulting in reduction of *E*. The ascending order of *U*_e_ is because that the larger the *σ*_p_/*σ*_ucs_, the greater the* m* values and axial strains, the areas between the unloading curve and *ε-*axis are thus larger. The phenomenon that *U*_irr_ increased as* m* value increased is probably due to the increment of the applied pressure stress, greater number of micro-cracks in non-uniform distribution area of microscopic failure stresses are formed, and these cracks caused more irreversible work was done. More discussion details of effect of *m* values on macroscopic physico-mechanical properties of specimens with different bedding dip angles can be found in following parts.

For specimens of group D, when *θ* = 0° (see Fig. 12a), *σ*_cd_/*σ*_ucs_, *ν*, *U*_e_ and *U*_irr_ (except 13, 24 and 30 loading cycles) were all in ascending order as *m* values increased, i.e., *σ*_cd_/*σ*_ucs_ were 0.61, 0.62, 0.63, 0.63, 0.65, 0.67, 0.68, 0.69, 0.71, 0.72, 0.73, 0.75, 0.75, 0.77, 0.77, 0.78, 0.79, 0.81, 0.81, 0.82 from cycles 12 to 31, the corresponding* m* values were 0.99, 0.99, 1.22, 1.34, 1.65, 2.04, 2.36, 2.62, 3.07, 3.65, 4.29, 5.27, 5.64, 6.69, 7.01, 8.13, 8.99, 9.92, 10.11, 10.33, which both were in ascending order. Following the same comparison procedure, Fig. 12b indicates that the relationship between* m* values and *σ*_cd_/*σ*_ucs_, *E*, *ν* and *U*_e_ of specimens of group D with *θ* = 60° were the same as that of* θ* = 0°, the larger the *m* value, the larger the *σ*_cd_/*σ*_ucs_, *ν* and *U*_e_, the smaller the *E*. However, *U*_irr_ decreased as *m* values increased. What’s more, the relationship between* m* values and *σ*_cd_/*σ*_ucs_, *E* (except 47 loading cycle), *ν* (except 47 loading cycle), *U*_e_ (except 47 loading cycles) and *U*_irr_ (except 47 loading cycles) of specimens of group D with *θ* = 90° were also the same as that of* θ* = 0°.

For specimens of group E, when *θ* = 0° (see Fig. 13a), the larger the *m* value, the larger the *σ*_cd_/*σ*_ucs_, *ν* and *U*_e_, the smaller the *E* (except 1 loading cycle). For example, *σ*_cd_/*σ*_ucs_ were 0.83, 0.85, 0.86, 0.87, 0.91, 0.90, 0.91, 0.91, 0.86 from cycles 1 to 9, the corresponding* m* values were 5.28, 7.13, 9.02, 10.31, 12.45, 13.02, 14.06, 15.17, 13.31. Figure 13a shows that *U*_irr_ decreased as *m* values increased for the first 4 loading cycles while it increased as *m* values increased in the following loading cycles. Following the same comparison procedure, Fig. 13b indicates that the relationship between* m* values and *σ*_cd_/*σ*_ucs_ (except 5 loading cycle), *E*, *ν*, *U*_e_ and *U*_irr_ of specimens of group E with *θ* = 60° were the same as that of* θ* = 0°. Figure 13c shows that for the first 6 loading cycles as *m* values in ascending order, *ν* (except 1 loading cycle), *U*_e_ and *U*_irr_ (except 1 loading cycle) were also in ascending order while *E* was in descending order as well as *σ*_cd_/*σ*_ucs_ was almost the same during these loading cycles except 1 cycle. For the cycles from 6 to 10, *σ*_cd_/*σ*_ucs_ and *E* decreased as *m* values decreased. Conversely, *ν*,* U*_e_ and *U*_irr_ were in ascending order during the same loading cycles as *m* values decreased.

### Effect of bedding dip angle on m values and macroscopic physico-mechanical properties

It can be observed from Fig. 10 that when the *σ*_p_/*σ*_ucs_ are the same (0.9–1.0), the *m* values of specimens of group D with *θ* = 0° and *θ* = 90°are almost the same which are greater than those of *θ* = 60°, indicating that distributions of microscopic failure stresses of specimens of group D with *θ* = 0° and *θ* = 90°are more homogeneous. While for the specimens of group E, the *m* values of *θ* = 0° are the largest, those of *θ* = 90° are the smallest, and those of *θ* = 60° are between them.

Some corresponding phenomenon that physico-mechanical properties affected by bedding dip angle are also can be observed from Figs. 7 and 9, i.e., *σ*_cd_,* σ*_cd_/*σ*_ucs_, *ε*_cd_, *ν*, *U*_e_ and *U*_irr_ are overall proportional to *m* values, the larger the* m* values, the greater these parameter properties, while for the *ε*_ucs_/*ε*_cd_,* E*, overall negative correlation between these parameter properties and *m* values can be observed. For example, for the *m* values and *σ*_cd_ of specimens of group D with *θ* = 0°and *θ* = 90°are both larger than those of *θ* = 60° (see Fig. 7a), which have positive relationship. *E* is taken as an example of the negative relationship*,* the *m* values of specimens of group E with *θ* = 60° are smaller than those of *θ* = 0°, but are greater than those of *θ* = 90°. What’s more, it is interested to note that some abnormal points can be observed in some case, this condition will be verified in further studies with more specimens.

The above results were reached based on a limited number of specimens, and they are applicable only to shale specimens at the laboratory scale to have a better understanding the dynamic evolution of the micro-structure homogeneity as well as their relationship to the physico-mechanical properties.

## Conclusions

Associated with a proposed equation between homogeneity index, *m*, and strain ratio, *ε*_ucs_/*ε*_cd_, Weibull distribution, a widely used statistical model characterizing the distribution of the microscopic physical–mechanical properties of rock or rock-like materials, was used to characterize the spatial variation of distribution of microscopic failure stress of shale specimens in laboratory scale subjected to dynamic load. Based on the experimental results of a series of cyclic loading tests on shale specimens with different bedding dip angles, the effect of the non-uniform distribution of microscopic failure stress on macroscopic physico-mechanical properties were analyzed. Some conclusions were apparent, and they are presented below:

1. Bedding dip angle affects the spatial distributions of microscopic failure stress of shale specimens subjected to dynamic load. The *m* values of specimens of group D (first-time unloading was carried out until the applied axial stress exceeded the *σ*_ci_ but lower than the *σ*_cd_) with *θ* = 0° and *θ* = 90°are almost the same which are greater than those of *θ* = 60°. While for the specimens of group E (first-time unloading was carried out when the applied axial stress higher than the *σ*_cd_), the *m* values of *θ* = 0° are the largest, those of *θ* = 90° are the smallest, and those of *θ* = 60° are between them.
2. The values of *σ*_cd_,* σ*_cd_/*σ*_ucs_, *ε*_cd_, *ν*, *U*_e_ and *U*_irr_ of the specimens with higher *m* values are overall higher while *ε*_ucs_/*ε*_cd_ and *E* are lower.
3. The *m* values have an ascending order during the dynamic load applying process with the increment of *σ*_p_/*σ*_ucs_, indicating that the dynamic load enables the spatial distributions of microscopic failure stress are more homogeneous prior to the final failure.
4. General speaking, *σ*_cd_/*σ*_ucs_, *ν*, *U*_e_ and *U*_irr_ increased as *m* values increased of each specimen. Conversely, *E* decreased as *m* values increased.
5. The final fracture morphology of specimens of both groups D and E (*θ* = 0°, *θ* = 60° and *θ* = 90°) were primarily controlled by the bedding surface.

## Supplementary Information

Supplementary Information.

## Data Availability

All data generated during this study are included in this published article [and its supplementary information files].
